# Cellular Islet Autoimmunity Associates with Clinical Outcome of Islet Cell Transplantation

**DOI:** 10.1371/journal.pone.0002435

**Published:** 2008-06-18

**Authors:** Volkert A. L. Huurman, Robert Hilbrands, Gabriëlle G. M. Pinkse, Pieter Gillard, Gaby Duinkerken, Pieter van de Linde, Petronella M. W. van der Meer-Prins, Minke F. J. Versteeg-van der Voort Maarschalk, Koen Verbeeck, Behrooz Z. Alizadeh, Chantal Mathieu, Frans K. Gorus, Dave L. Roelen, Frans H. J. Claas, Bart Keymeulen, Daniel G. Pipeleers, Bart O. Roep

**Affiliations:** 1 Department of Immunohematology and Blood Transfusion, Leiden University Medical Center, Leiden, The Netherlands; 2 Department of Surgery, Leiden University Medical Center, Leiden, The Netherlands; 3 Diabetes Research Center, Brussels Free University-VUB, Brussels, Belgium; 4 Laboratory for Experimental Medicine & Endocrinology (LEGENDO), University Hospital Gasthuisberg, Catholic University of Leuven-KUL, Leuven, Belgium; 5 JDRF Center for Beta Cell Therapy in Diabetes, Brussels, Belgium; The National Institute of Diabetes and Digestive and Kidney Diseases, United States of America

## Abstract

**Background:**

Islet cell transplantation can cure type 1 diabetes (T1D), but only a minority of recipients remains insulin–independent in the following years. We tested the hypothesis that allograft rejection and recurrent autoimmunity contribute to this progressive loss of islet allograft function.

**Methodology/Principal Findings:**

Twenty-one T1D patients received cultured islet cell grafts prepared from multiple donors and transplanted under anti-thymocyte globulin (ATG) induction and tacrolimus plus mycophenolate mofetil (MMF) maintenance immunosuppression. Immunity against auto- and alloantigens was measured before and during one year after transplantation. Cellular auto- and alloreactivity was assessed by lymphocyte stimulation tests against autoantigens and cytotoxic T lymphocyte precursor assays, respectively. Humoral reactivity was measured by auto- and alloantibodies. Clinical outcome parameters - including time until insulin independence, insulin independence at one year, and C-peptide levels over one year- remained blinded until their correlation with immunological parameters. All patients showed significant improvement of metabolic control and 13 out of 21 became insulin-independent. Multivariate analyses showed that presence of cellular autoimmunity before and after transplantation is associated with delayed insulin-independence (p = 0.001 and p = 0.01, respectively) and lower circulating C-peptide levels during the first year after transplantation (p = 0.002 and p = 0.02, respectively). Seven out of eight patients without pre-existent T-cell autoreactivity became insulin-independent, versus none of the four patients reactive to both islet autoantigens GAD and IA-2 before transplantation. Autoantibody levels and cellular alloreactivity had no significant association with outcome.

**Conclusions/Significance:**

In this cohort study, cellular islet-specific autoimmunity associates with clinical outcome of islet cell transplantation under ATG-tacrolimus-MMF immunosuppression. Tailored immunotherapy targeting cellular islet autoreactivity may be required. Monitoring cellular immune reactivity can be useful to identify factors influencing graft survival and to assess efficacy of immunosuppression.

**Trial Registration:**

Clinicaltrials.gov NCT00623610

## Introduction

Islet cell transplantation has considerable potential as a cure for type 1 diabetes (T1D) [1]. In 2000, a cohort of seven patients remained insulin-independent for one year after transplantation under a steroid-free immunosuppressive regimen [2]. Several groups have reported similar short-term success, using different islet isolation and immunosuppressive regimens [3]–[5]. The procedure seems safe and is associated with low morbidity [6], but long-term insulin independence is rare [7].

At present, a major challenge is to determine which factors influence graft survival [8]. Variables studied usually relate to the transplantation procedure (isolation method, culture, transplantation technique, quality and quantity of the graft), the engraftment (impaired revascularization [9], apoptosis [10], β-cell exhaustion [11], donor characteristics) and the immunosuppressive treatment [12]. We recently demonstrated that the β-cell mass injected correlated significantly with metabolic outcome at posttransplant month 2 [13]. Other factors are also expected to influence short- and long-term function of islet grafts, but their identification is difficult in view of the variability in donor and recipient characteristics in islet transplant protocols. The methods used in our clinical study [3], [13] allow to standardize donor tissue for cellular composition and beta cell mass [3] and thus facilitate further analysis of immune factors. They should help examine whether signs of islet cell auto- and alloreactivity in recipients affect successful clinical outcome independently of graft related variables.

T1D is an autoimmune disease characterized by T cell mediated destruction of β-cells, in which CD4+ T helper cells seem to play a pivotal role [14], [15]. It can thus be anticipated that success of β-cell replacement not only requires suppression of allograft rejection, but also prevention of a recurrent T-cell mediated autoimmune process, as has been demonstrated in experimental models [16], [17]. Autoantibody seroconversion has been considered as a sign of recurrent autoimmunity after whole pancreas [18] and β-cell transplantation [19]–[21], but this is not a consistent finding [3]. Although diabetes-associated autoantibodies are important as diagnostic markers of preclinical T1D [22], [23], there is no direct evidence for their role in the pathogenesis of the disease [24], [25]. Consequently, islet autoantibodies have proved to be of limited value in immune monitoring of intervention or islet transplantation [25], even though correlations between pre-transplant autoantibody status and outcome have been reported [26].

In the past, we have developed reproducible methods for quantification of both antigen-specific cellular autoreactivity and allograft-specific cellular cytotoxicity [27]–[30]. The main aim of this study was to combine these methods with established methods for HLA- and autoantibody detection [31], [32], to identify immune markers for successful β-cell transplantation in the same cohort of islet graft recipients that we reported on earlier and that were transplanted in a standardized protocol [13].

## Methods

### Transplantation and clinical follow-up

**Figure 1:**
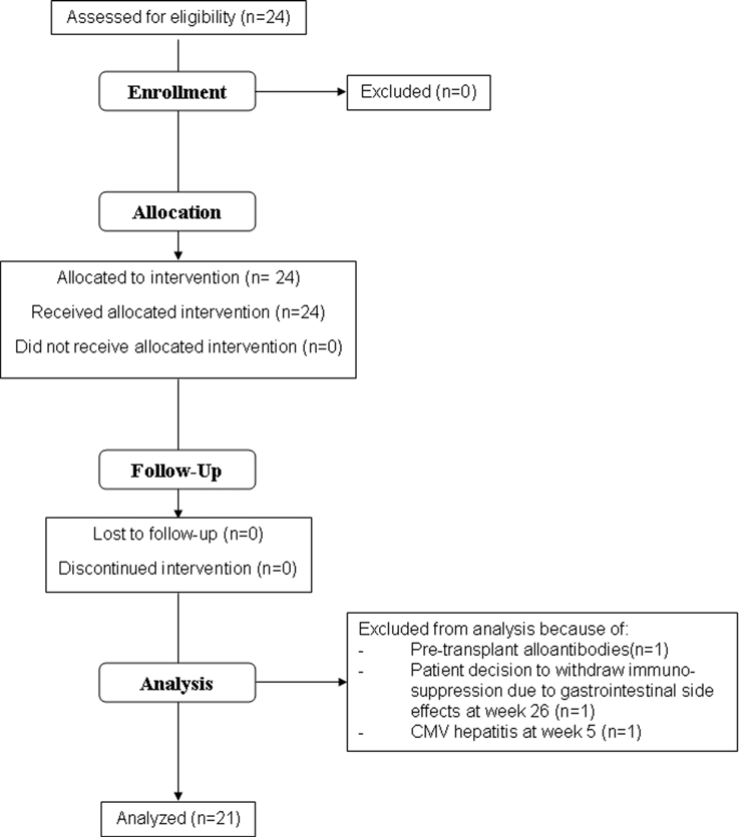
CONSORT-style flowchart of 24 consecutive islet cell transplantation recipients.

the protocol for this trial and supporting CONSORT checklist are available as supporting information; see CONSORT S1 and Protocols S1. Twenty-four consecutive patients were transplanted with one (n = 10) or two (n = 14) islet cell grafts with 1–6 donors per graft (4 donors median) after signing informed consent and under appropriate ethical approval. As we reported previously [13], two patients were lost to follow-up in the first year, one due to CMV infection and another due to withdrawal of consent. Before transplantation, one of the twenty-two remaining patients presented alloantibodies against HLA alloantigen that was expressed on the donor cells. As pre-immunization to alloantigens is an established predictor of poor graft survival [33], this patient was excluded from the current analysis (Figure 1). Relevant baseline patient characteristics are shown in Table 1. Total number of donors per patient ranged from 2 to 10 (6 median). Graft recipients were long-term type 1 diabetes patients without any earlier transplantation, with plasma C-peptide <0.09 ng/ml, large variation in blood glucose levels (Coefficient of variation [CV] ≥25%), HbA_1c_ concentration>7% and one or more chronic diabetes lesions. Exclusion criteria were: body weight>90 kg, active smoking, pregnancy, disturbed liver function tests, history of hepatic disease, presence of HLA antibodies or negative EBV serostatus.

**Table 1:** Recipient characteristics

Parameter	Median ( IQR) N = 21
Age (yr)	42 (37–49)
Gender (M/F)	13/8
Body weight (kg)	69 (65–76)
Duration of disease (yr)	26 (19–33)
Age at onset (yr)	17 (12–24)
HbA1c (%)	7.6 (6.9–8.1)
Insulin dose (IU/kg/d)	0.7 (0.5–0.9)
Mean fasting glycemia (mg/dl)	174 (145–195)

Donor organs were procured from multiple heart-beating donors through the Eurotransplant Foundation (Leiden, The Netherlands) and processed at the Beta Cell Bank in Brussels to beta cell enriched fractions that were cultured for 2–20 days (median 6 days). The grafts were characterized by their cellular composition – in particular the number and purity of insulin-containing beta cells [13]. They were injected into the portal vein of the recipient as previously described [3], [6], [13].

Immunosuppressive induction therapy consisted of anti-thymocyte globulin (ATG, Fresenius, Fresenius Hemocare, WA, USA) with a single infusion of 9 mg/kg and subsequently with 3 mg/kg for 6 days except when T-lymphocyte count was under 50/mm^3^. Maintenance immunosuppression consisted of tacrolimus (Prograft, Fujisawa/Pharma Logistics, dose according to trough level: 8–10 ng/ml in the first three months post transplantation, 6–8 ng/ml thereafter) and mycophenolate mofetil (MMF, Roche, 2000 mg/day).

Graft recipients were regularly followed up for 1 year regarding plasma C-peptide levels at glycemia 120–200 mg/dl (weekly during the first months, monthly thereafter) and HbA_1c_ concentration. The C-peptide level over one year was calculated by the area under the curve (AUC) of available plasma C-peptide values in the first year after transplantation.

The decision to inject a second β-cell graft was based on the C-peptide levels and/or variation of fasting glycemia (CV glucose>25%) after the first engraftment [13]. Insulin tapering was only considered in patients with plasma C-peptide values≥1.0 ng/ml (glycemia 120–200 mg/dl), CV fasting glycemia <25% and mean fasting glycemia <125 mg/dl. It was started after month 2 at a rate of minus 2IU every 3–5 days unless patients presented hypoglycaemic episodes (<70 mg/dl). Insulin treatment was reintroduced after two consecutive HbA_1c_ measurements>7.0% (HbA_1c_ was measured bi-monthly during the first 6 months and monthly afterwards).

### Lymphocyte Stimulation Test to determine cellular autoreactivity

All cellular reactivity tests were performed blinded from clinical results. Blood was drawn from patients before transplantation and on regular intervals post transplantation (standard once every two weeks in the first months post transplant and once every two months until one year.). Peripheral blood mononuclear cells (PBMCs) were isolated and processed as described before [30]. Briefly, 150.000 fresh PBMCs/well were cultured in 96 well round-bottomed plates in Iscove's Modified Dulbecco's Medium (IMDM) with 2 mMol/l glutamine (Gibco, Paisley, Scotland) and 10% pooled human serum in the presence of antigen, IL-2 (35 U/ml) or medium alone in triplicates. After 5 days ^3^H-thymidine (0.5 μCI per well) was added for 16 hours and ^3^H-thymidine incorporation was measured. Antigens analyzed included IA-2 (10 μg/ml), GAD65 (10 μg/ml), insulin (25 μg/ml) and tetanus toxoid (‘third party’ antigen, 1,5 LF/ml). Results were interpreted as stimulation index (SI) compared to medium value, where an SI>3 was considered positive. After transplantation, positivity in case of incidental SIs between 3 and 5 was defined based on the pattern and frequency of cellular autoreactivity over time, blinded from clinical outcome.

### Cytotoxic T lymphocyte precursor (CTLp) assay to determine the number of alloreactive T cells

The CTLp assay has been described in detail previously [27]. Briefly, cryopreserved PBMCs from recipients from before and different time points after transplantation were cultured in a limiting dilution assay (40.000 to 625 cells/well, 24 wells per concentration) with different irradiated stimulator PBMCs expressing HLA class I antigens that are also expressed on the injected β-cell grafts (50.000 cells/well, 3 to 8 different stimulators depending on the number of donors and mismatches). Cells were cultured for seven days at 37°C in 96-well round-bottomed plates in RPMI 1640 medium with 3 mM L-glutamine, 20 U/ml IL-2 an 10% pooled human serum. Next, Europium-labelled graft HLA-specific target cells (5.000 cells/well, 4 to 8 different targets) were added to the stimulator/responder combinations for 4 hours. Wells were scored positive if the Europium release through target cell lysis exceeded spontaneous release +3 SD. Quantification of CTLp frequencies was performed by computer software developed by Strijbosch *et al*. [34]. Cytotoxic alloreactivity in the first year after transplantation was analyzed blinded from clinical outcome and classified as either low or increased, based on the CTLp frequencies against the different mismatch combinations and their evon over time.

### Autoantibodies

All available samples were tested for islet cell autoantibodies (ICA), autoantibodies against IA-2 protein (IA-2A) and glutamate decarboxylase (GADA), as described before [32]. Briefly, ICA were determined by indirect immunofluorescence and end-point titers expressed as Juvenile Diabetes Foundation (JDF) units. IA-2A and GADA were determined by liquid phase radiobinding assays, and expressed as percent tracer bound. Cutoff value determination was described before [32], and amounted to ≥12 JDF units for ICA, ≥2,6% for GADA and ≥0,44% for IA-2A.

Post transplant seroconversion was determined as the appearance of autoantibodies which were not detectable before transplantation or disappearance of previously detectable autoantibodies during the first year following transplantation.

### Anti-HLA antibodies

Patient sera were screened for the presence of HLA class I and class II specific antibodies by ELISA (LAT class I & II, One Lambda, CA). When positive, the specificity of HLA Class I antibodies was determined by complement-dependent cytotoxicity assay against a selected panel of 52 HLA typed donors.

### Statistics

Univariate analysis of time to insulin independence was performed by Kaplan Meier analysis, using the log rank test to assess significance. Analysis of dichotomous data was performed by Fischer exact test and χ^2^ test. Quantitative differences between groups were analyzed by unpaired t-test and non-parametric Mann-Whitney U test as well as one-way ANOVA.

For multivariate analyses, Cox proportional hazards regression was used to assess time to insulin independence, binary logistic regression to assess insulin independence at one year, and multiple linear regression to determine differences in total C-peptide levels. Multivariate analysis was performed in a stepwise fashion with the p-value for entry into or removal from the analysis set at 0.20, to allow for inclusion of variables tending towards significance in this analysis of a relatively limited number of patients. Analyses were performed using GraphPad Prism (version 4.0) and SPSS (version 14.0) software. P<0.05 was considered significant.

## Results

### Transplants and metabolic outcome

For the 21 patients studied in the current analysis, median total β-cell mass injected was 3.9×10^6^ (Interquartile range [IQR] 2.9×10^6^ –5.0×10^6^) cells/kg body weight. Median β-cell mass per transplant was 2.4×10^6^ (IQR 1.7×10^6^–3.1×10^6^) million cells/kg body weight. (Transplant related parameters per patient are available in Table S1.)

All patients showed significant improvement of metabolic control. Out of the 21 patients examined, 20 (95%) showed β-cell function (defined as plasma C-peptide≥0.5 ng/ml) at any time point in the first year after transplantation. Thirteen patients (62%) achieved insulin independence. Three of these patients resumed insulin therapy within one year after first transplantation [13].

### Serology

Allo- and autoantibody data were available from all patients. Of 22 patients transplanted under the current protocol, one patient was excluded from the current analysis because of pre-sensitization with graft-specific HLA antibodies (see Methods section). No HLA antibodies were observed in any of the other patients during the course of this study.

Six out of 21 patients were positive for at least two islet autoantibodies (ICA, GADA and IA-2A) before transplantation. Ten patients were positive for a single autoantibody. One of the three patients who developed new autoantibodies after transplantation reached insulin independence, and two other patients who lost an autoantibody reactivity both became insulin-independent. (Detailed and individual immune-related parameters are available in Table S1.)

### Cellular reactivity

Complete data on cellular autoreactivity could be obtained from 18 out of 21 patients. Of one patient, no pre-transplantation assessment of autoreactivity was performed for logistic reasons. In two other cases, data on reactivity to IA2 was lacking due to temporary unavailability of the recombinant IA2 antigen. Cellular islet autoreactivity against GAD and/or IA-2 was detected in 10 patients (56%) before transplantation, four of them being reactive to both autoantigens GAD and IA-2, three against GAD and three against IA-2 only. Cellular reactivity to whole insulin protein remained low in all patients both before and after transplantation; therefore the response to whole insulin was excluded from the analysis. Four out of ten patients retained cellular autoreactivity after transplantation. Among the eight patients without detectable cellular autoreactivity before transplantation, five developed it post transplantation. Incidental moderate cellular autoreactivity (3<SI<5) was detected in eight patients after transplantation; five of these cases were interpreted as negative on basis of the pattern in time, while three cases were judged positive in view of their repeatedly increased cellular autoreactivity.

Alloreactive CTL precursor analysis determining donor HLA-specific cellular cytotoxicity was performed in 20 out of 21 patients. The total number of donors per patient ranged from 2 to 10 (mean of 6), representing 9 to 29 (mean 18) HLA class I mismatches per patient. Using extensive mismatch combinations and large HLA panels, on average 78% of mismatches could be evaluated per patient. By this analysis of alloreactivity, for 97% of the grafts at least part of the HLA mismatches with the recipient were covered. For 60% all of the grafts' mismatches were covered. Nine patients (45%) developed islet donor-specific alloreactive cytotoxicity over one year, as indicated by the CTLp assay.

### Association of immunological parameters with clinical outcome

To identify possible predictors for transplant outcome, the immunological parameters were analyzed with respect to three clinically relevant endpoints: time to insulin-independence, insulin-independence at one year, and C-peptide level over one year. The immune parameters included immune suppressive therapy (Tacrolimus trough levels, ATG and MMF dosage), pre-transplant cellular autoreactivity, post-transplant cellular autoreactivity, post-transplant donor HLA-specific cellular cytotoxicity, presence of pre-transplant autoantibodies, post-transplant autoantibody seroconversion. Injection of sufficient β-cell mass (proposed earlier as ≥2.0×10^6^ β-cells per kg body weight per injection [13]) was also analyzed.

**Figure 2:**
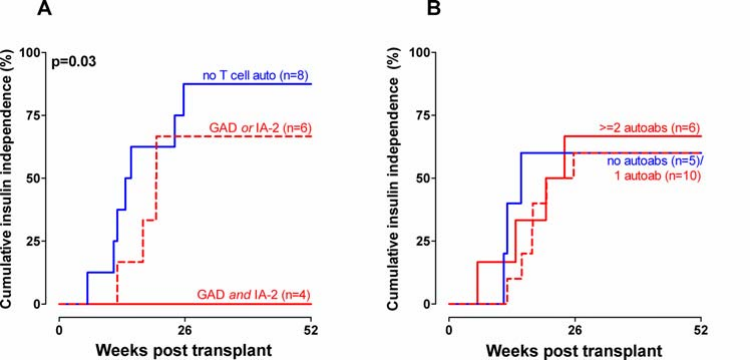
Kaplan-Meier curves showing cumulative insulin independence after β-cell transplantation, stratified for A) pre-transplant cellular autoimmunity and B) pre-transplant presence of autoantibodies. Continuous lines represent patients without reactivity to autoantigens, striped lines patients with reactivity to a single antigen, and dotted lines patients with reactivity to two antigens (or three in the case of autoantibodies).

Pre-transplant cellular autoreactivity was associated with delayed achievement of insulin-independence (overall χ^2^ = 6.91, p = 0.03). The extent of pre-transplant cellular autoreactivity was of additional influence, as patients reactive to both GAD and IA-2 never reached insulin-independence (log rank: χ^2^ = 6.49, p = 0.01 vs. non-autoreactive patients, Figure 2A), whereas patients reactive to a single islet autoantigen did so in four out of six cases, (χ^2^ = 3.74, p = 0.05 for time to insulin independence when compared to double-positive patients). No such influence was observed regarding the presence of pre-transplant autoantibody production (Figure 2B). Tacrolimus trough level and insufficient injected β-cell mass was also associated with delayed insulin-independence (p = 0.04 and p = 0.02, respectively, Table 2). In multivariate analysis, both pre- and post transplant cellular autoreactivity were significantly associated with delayed insulin independence (p = 0.001, Relative Risk (RR) 0.133 [0.039–0.453]) and p = 0.01, RR 0.224 [0.147–0.892]), respectively). None of the four patients reactive against both IA-2 and GAD insulin became independent, whereas two out of six ‘single’ cellular autoreactive patients (33%) and six out of eight non-autoreactive patients (75%) were insulin independent at one year (Fischer exact p = 0.06). (Quantitative pre-transplant Stimulation Indices against GAD and IA-2 for patients reaching or not reaching insulin independence are shown in Figure S1.)

**Table 2:** Impact of immune parameters on clinical outcome after islet cell transplantation.

Endpoint		Time to insulin independence					Insulin independence at one year					AUC of plasma C-peptide over one year				
		Univariate*			Multivariate**		Univariate†			Multivariate††		Univariate‡			Multivariate‡‡	
Variable		I.I.	χ^2^	p	R.R. (95%C.I.)	p	I.I.	χ^2^	P	R.R. (95%C.I.)	p	Mean (±S.E.)	χ^2^	P	Beta (95%C.I.)	p
ATG dosage		13/21	1.63	0.20		ns	10/21	Z = −0.39	0.71		ns	82.51 (±8.28)	−0.33	0.15	−3.95 *(*−*7.70 to* −*0.21)*	**0.04**
**Tacrolimus trough level^+^**		“	4.13	**0.04**		ns	“	Z = −0.96	0.35		ns	“	0.46	**0.04**		ns
MMF dosage		“	0.00	1.00		ns	“	Z = −0.30	0.81		ns	“	0.02	0.94		ns
**Pre-transplant cellular autoreactivity**	0	7/8	6.91	**0.03**	0.133 *(0.039 to 0.453)*	**0.001**	6/8	6.53	**0.04**	0.025 *(0.001 to 1.241)*	0.06	109.89 (±10.18)	F = 8.07	**0.004**	−26.73 *(*−*41,76 to* −*11,70)*	**0.002**
	1	4/6					2/6					77.72 (±11.77)				
	2	0/4					0/4					39.96 (±14.30)				
Pre-transplant auto-antibodies	0	3/5	0.28	0.87	0.440 *(0.171 to 1.136)*	0.09	2/5	0.15	0.93		ns	97.19 (±23,73)	F = 0.86	0.44		ns
	1	6/10					5/10					71.50 (±10.89)				
	≥2	4/6					3/6					88.61 (±12,03)				
**Post-transplant cellular autoreactivity**	0	7/9	2.10	0.35	0.224 *(0.147 to 0.892)*	**0.01**	5/9	1.19	0.55	0.161 *(0.013 to 2.033)*	0.16	91.65 (±10.83)	F = 0.35	0.71	−21.01 *(*−*37.65 to* −*4.42)*	**0.02**
	1	3/7					2/7					75.28 (±19.60)				
	2	1/2					1/2					76.73 (±.4.32)				
Post-transplant auto-antibody seroconversion	−	2/2	2.82	0.24		ns	2/2	2.54	0.28		ns	109.99 (±21.61)	F = 1.79	0.20		ns
	=	10/16					7/16					85.16 (±9.03)				
	+	1/3					1/3					50.06 (±24.07)				
Post-transplant cellular alloreactivity	−	7/11	0.09	0.76		ns	5/11	n/a	1.00		ns	71.90 (±10.90)	t = −1.24	0.23		ns
	+	5/9					4/9					93.04 (±13.41)				
**All injections ≥ 2.0×10^6^ β-cells/kg**	−	2/7	5.65	**0.02**		ns	1/7	n/a	0.06		ns	50.24 (±10.28)	t = −3.41	**0.003**	27.46 *(3.64 to 51.22)*	**0.03**
	+	11/14					9/14					98.64 (±8.58)				

Shown are univariate and stepwise multivariate analyses for time to insulin independence (A), Insulin independence at one year (B), and total C-peptide level over one year (C). Parameters significantly associated with outcome are depicted in **bold**.

* logrank test; ^**^ Cox proportional hazard regression; ^†^ Mann-Whitney U test/Fischer's exact test/χ^2^ test; ^††^ logistic regression; ^‡^ Pearson's correlation/unpaired t-test/one-way ANOVA; ^‡‡^ linear regression; ^+^ average Tacrolimus trough level in months 0–3 analyzed for endpoint A, in months 0–12 for endpoint B and C. When more than two groups are analyzed, the univariate columns refer to overall p-values. I.I.:insulin independence; R.R.: relative risk; C.I.: confidence interval; S.E.: standard error.

C-Peptide level over one year was strongly associated with cellular autoreactivity (p = 0.004, Table 2), with similar baseline characteristics and injected β-cell mass for the three groups. Tacrolimus trough level (p = 0.04) and graft size (p = 0.003) also influenced C-peptide level univariately. In multivariate analysis, both pre-transplant (p = 0.002, beta −26.73 [−41.76 to −11.70]) and post-transplant cellular reactivity (p = 0.02, beta −21.01 [−37.65 to −4.42]), as well as graft size (p = 0.03, beta 27.46 [−3.64 to 51.22]) and ATG dosage (p = 0.04, beta −3.95 [−7.70 to −0.21]) were significantly associated with C-peptide level over the first year (Table 2).

Neither MMF dosage nor pre-transplant autoantibody status or post transplant seroconversion affected time to insulin independence, insulin-independence at one year or C-peptide level over one year (Table 1). Similarly, cellular cytotoxicity against the HLA-allodeterminants of the islet donors did not correlate with any of these three clinical endpoints.

### Influence of cellular autoreactivity over time

**Figure 3:**
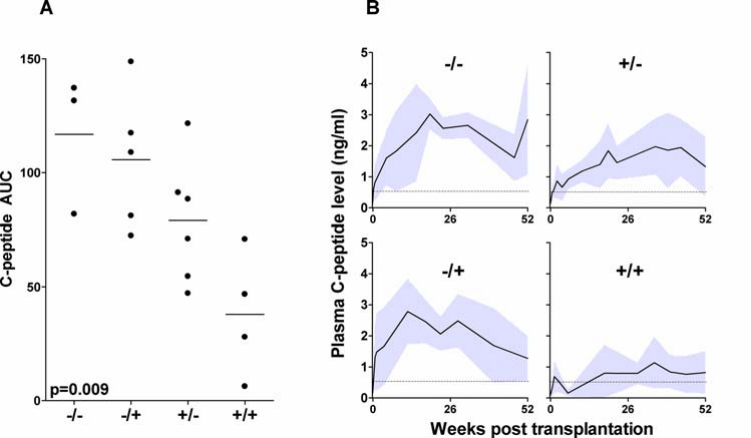
A) C-peptide levels stratified for cellular autoimmune status before and after transplantation. Total C-peptide levels over one year for patients that are not autoreactive pre- nor post-transplant (−/−, n = 3) only pre- (+/−, n = 6) or only post-transplant (−/+, n = 5), and both pre- and post transplant (+/+, n = 4). Areas under the curve differ significantly between groups (p = 0.009, one-way ANOVA). Horizontal lines represent average C-peptide level per group. B) average basal C-peptide levels (black lines)±SD (grey areas) over time for the four different groups. Differences between −/− and +/+ and between −/+ and +/+ remain significant after Bonferroni correction. Pre-transplant autoreactivity significantly reduces total C-peptide production (p = 0.006, unpaired t-test).

The combined influence of pre-and post-transplant cellular autoimmunity on graft function was assessed by separating the patients into four groups: cellular autoreactivity against one or more autoantigens before and after last transplantation (+/+),before transplantation only (+/−), development of cellular autoreactivity after last transplantation (−/+), and no cellular autoreactivity at any time point (−/−). Time to insulin-independence was associated with the cellular autoreactivity status before and after islet transplantation (overall χ^2^ = 8.69, p = 0.03 by log rank test, χ^2^ = 5.93 and p = 0.01 for trend). Insulin independence was reached in 0 out of 4 patients in the +/+ group, 4/6 in the +/− group, 4/5 in the −/+ group and 3/3 in the −/− group. Injected β-cell mass was similar between the groups. C-peptide level over the first year differed between the four groups (Figure 3, R^2^ = 0.55, p = 0.009). The linear trend for all four groups was highly significant (R^2^ = 0.48, p = 0.002). Differences between −/− and +/+ and between −/+ and +/+ remained significant after Bonferroni adjustment. Plasma C-peptide level over 52 weeks was mostly affected by pre-transplant autoreactivity (R^2^ = 0.39, p = 0.006 by unpaired t-test). Even in the first six weeks (before any second implantation) this pattern was observed (R^2^ = 0.36, p = 0.008 comparing C-peptide AUC in week 0–6).

### Influence of pre-transplant autoreactivity is confined to patients with low injected β-cell mass

**Figure 4:**
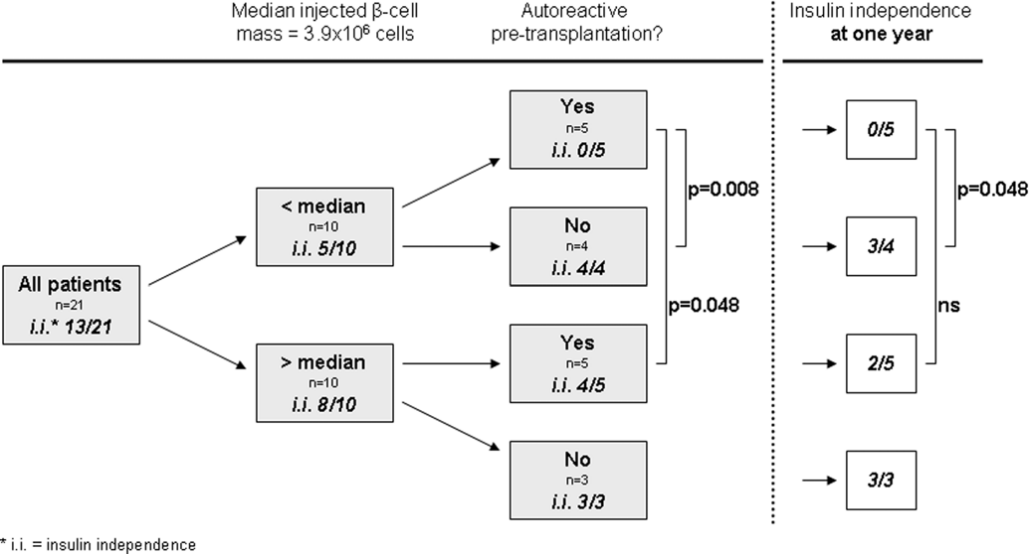
Influence of pre-transplant T cell autoreactivity stratified for total injected β-cell mass. Shown are pre-transplant T cell autoreactivity and achievement of insulin independence for patients receiving more or less than the median total injected β-cell mass (the single patient receiving the median β-cell mass is excluded). Groups are compared by Fischer exact test.

Pre-existent cellular autoreactivity was further studied in relation with injected β-cell mass that was previously shown to be indicative for clinical transplant success [13]. The influence of pre-existent cellular autoreactivity on insulin-independence was confined to the subgroup of patients receiving amounts of β-cells lower than the median (Fischer exact p = 0.008, Figure 4). This effect lasted during the entire follow-up. Within the patients receiving more β-cell mass than the median, no significant influence of cellular autoreactivity was seen. Additionally, patients with pre-existent islet autoimmunity reached insulin independence less frequently when they had received less than the median of β-cell mass compared to those receiving more beta-cells (Fischer exact p = 0.048), although this difference did not persist at one year post transplant.

## Discussion

The aim of this study was to identify immunological correlates for islet transplant survival. Where we and others have described markers of allo-and autoimmunity after islet cell transplantation [3], [18]–[21], [28], [33], [35], [36], our present results have possible implications for the selection and treatment of type 1 diabetic candidate islet recipients before transplantation. Despite the limited number of patients studied that can be studied in clinical islet transplant trials, significant associations of clinical outcome with immunological parameters were derived from blind analysis of data in a group of 21 islet cell recipients. When interpreting results, some caveats need to be kept in mind. We report an exploratory study using assays for cellular autoreactivity that have proved difficult to perform and shown variability between institutions in the past [37]. However, a structured approach validating our techniques has been implemented by the T Cell Workshop of the Immunology of Diabetes Society founded and directed by our institute, that provided considerable experience and reproducibility [28]–[30], [37], [38]. Second, the limited number of patients inherent to clinical islet transplantation trials combined with a large number of immune variables studied enforced us to apply stepwise multivariate analysis to assess the independence of new predictors. This may be subject to debate because of the increased possibility of reporting chance findings. However, while full multivariate analysis may be preferable, the current analysis optimizes study power and allows for detection of valuable markers which otherwise may not have been identified in this exploratory study.

In this patient group receiving grafts with standardized cellular composition, the injected β-cell mass was shown to be an important variable for clinical outcome at month 2 after implantation [13]. We now demonstrate that cellular autoimmune reactivity before transplantation is another variable that is associated with achievement of insulin independence, as well as AUC of C-peptide level over 52 weeks. Obviously, these results will need further confirmation in larger patient cohorts with longer follow-up. Furthermore, non-immunological factors could influence transplant survival as well and need to be assessed in future studies.

Outcome is significantly worse in patients who showed cellular autoreactivity prior to transplantation. This finding is unexpected, since diabetes-associated islet autoantigens are thought to be lost in these long-term type 1 diabetes patients prior to transplantation [39]. These pre-existent autoreactive T cells may cause rapid initial destruction of beta cells as is suggested by the increased need for a second infusion (8/10 vs. 2/8 in non-autoreactive patients).

Interestingly, pre-existing GAD-specific autoreactivity in the peripheral blood samples disappeared after transplantation in all patients, whereas IA-2-specific autoreactivity frequently persisted. GAD is also expressed by other tissues and therefore continuously present as an autoantigen in the body. This notion implies that immunological memory of autoreactivity is exerted differentially between autoantigens and may therefore be affected differently by immunosuppression. No cellular autoreactivity was observed to whole insulin protein during follow-up, while insulin is considered one of the major autoantigens in T1D. This lack of responsiveness may result from many years of therapy with exogenous insulin, or insufficient antigen processing and presentation. Indeed, we did observe occasional reactivity when a specific insulin epitope (e.g. B9-23) was tested . However, insulin epitope data were only available for a fraction of our study population.

Eighty-eight percent of recipients lacking pre-transplant cellular autoreactivity became insulin independent after transplantation, with 75% remaining off insulin therapy at one year. These short-term results are in the same range as those achieved in whole organ pancreas transplantation [40]. Plasma C-Peptide level (calculated by AUC over one year) gives a complete overview of graft function, incorporating both peak and duration of C-peptide production. C-peptide level also showed significant association with cellular autoreactivity. Still, although none of the patients with positivity to both GAD and IA2 before transplantation became insulin independent, some showed considerable C-peptide level (Table S1). To assess the influence of a possible second transplant, the C-peptide level before any second transplant (AUC from 0 to 6 weeks) was calculated, showing similar results.

Cellular reactivity to autoantigens after islet cell transplantation did not correlate with graft function in univariate analysis. However, in combination with pre-transplant autoreactivity, post-transplant autoreactive status becomes informative, as underscored by its significance in multivariate analysis. However, recurrence of autoimmunity that was undetectable prior to transplantation could represent a different process than pre-existing autoreactivity and it is conceivable that the role of recurrent autoimmunity may become more apparent after longer follow-up.

The presence of autoreactive CD8^+^ T-cells in islet graft recipients losing graft function supports a particular role for recurrent autoimmunity after islet transplantation [41]. Some patients became insulin-independent in spite of developing islet autoreactivity after transplantation, indicating that additional mechanisms may be able to rescue graft function. In experimental models, recurrent autoimmunity after islet transplantation has been abrogated successfully [16], [17], but such success has not yet been reported in humans.

In pancreas transplantation, recurrent autoimmunity after transplantation has been reported [18]–[20], [42], [43], but was mainly limited to the presence of autoantibodies, that in one study have shown impact on pancreas transplantation survival rates [44]. In the whole pancreas transplantation program in our institute (>90% one-year graft survival), we observed only very limited post-transplant cellular autoreactivity. However, pre-transplant cellular autoimmunity was not studied in these patients. Differences with our results in islet transplantation include the protective or regenerative capacity of the non-islet pancreatic tissue, vascularization and the smaller islet cell mass in injected islets than in whole pancreas transplants.

Changes in islet autoantibody status did not qualify as an independent surrogate marker for β-cell survival in this cohort. This is in accordance with several studies describing a lack of their association with clinical remission or therapeutic intervention [25], but discord with earlier claims in islet or pancreas transplantation [18]–[20], [45]. It is conceivable that seroconversion may be a surrogate marker for (loss of) β-cell function in some cases, but it does not appear to reflect the primary autoimmune process influencing transplant success. If larger series would indicate that islet autoantibodies are associated to loss of islet graft function, our data suggest that this is secondary to T-cell autoreactivity.

Occurrence of, or pre-sensitization with, graft-specific alloantibodies is a known risk factor for transplant failure [33] but was exceptional in our cohort. This is an important consideration in view of the fact that these T1D patients, despite islet cell transplantation, may need a kidney transplantation for diabetic nephropathy in a later stage.

Perhaps surprisingly, T-cell cytotoxicity to alloantigens on the islet grafts was not independently associated with clinical outcome. Yet, alloreactivity against islets served as most frequent correlate with graft failure in islet-after-kidney transplantation, as we reported earlier [28]. Differences between islet transplantation alone versus islet-after-kidney transplantation include preconditioning of the patients with immune suppression, the type of immune suppression (tacrolimus and MMF vs. prednisolone, cyclosporine and azathioprine, respectively) and a history of successful implantation of a kidney allograft years before. Development of alloreactive CTLs did not lead to production of graft–specific HLA antibodies in our protocol.

Explanations for the lack of correlation between alloreactivity and islet allograft function may further relate to the notion that the CTLp assays employed were not designed to distinguish between high and low avidity of the T-cell receptor, where elevated frequencies of low-avidity CTLs need not be detrimental. The presence of effective immunosuppressive therapy may be of additional influence in this context. Indeed, our preliminary experiments indicate that addition of tacrolimus in the CTLp assay suppressed allo-CTLp frequencies in a successfully transplanted patient, whereas allo-CTLp frequencies in a case of graft failure remained elevated. Second, the influence of alloimmunity may be secondary to a preimmunized autoimmune status. In this scenario, alloreactivity may be precipitated by an initial autoimmune attack to the islet allograft, leading to so-called determinant spreading that includes alloantigens [46]. Finally, the current immunosuppressive regimen that is largely based on prevention of allograft rejection may cope sufficiently with *de novo* T-cell alloreactivity, but may prove inadequate to intervene in (pre-existent) islet autoreactivity. The latter interpretation is supported by earlier findings that immunosuppressive therapy (e.g., prednisolone, cyclosporin A, azathioprine) had at most temporary effects on preservation of β-cell function at clinical manifestation of type 1 diabetes [47]–[49].

In conclusion, our results indicate that cellular autoimmunity may influence survival of islet cell allografts in type 1 diabetic recipients. The amount of autoreactivity (to one or two antigens) has additional influence, implying a role in patient selection. Obviously, longer follow-up and enlargement of patient cohorts will be warranted to confirm these findings. Yet, the combination of sufficiently large β-cell mass and a non-autoreactive recipient currently appears the most desirable condition to perform successful β-cell transplantation. As suggested earlier [8], longitudinal analysis of auto- and alloreactivity may be a useful tool to identify immune factors influencing graft survival and to assess efficacy of immunosuppression. We propose that tailoring immunosuppressive treatment of islet autoreactivity, as well as adjusting graft size for individual patients, can improve clinical outcome after islet cell transplantation.

## Supporting Information

Table S1 Characteristics of immune reactivity and clinical parameters per patient.
(0.14 MB DOC)

Figure S1 Stimulation Indices of pre-transplant proliferation against GAD (grey) or IA-2 (black) of patients that were insulin independent at one year (A); patients reaching insulin-independence that was lost before week 52 (B); and patients that never reached insulin-independence (C). (D) Stimulation Indices per group. Insulin-dependent and -independent patients were compared by Mann Whitney U test. (Shown are median + interquartile range.)
(0.99 MB TIF)

Checklist S1 CONSORT Checklist.
(0.06 MB DOC)

Protocol S1 Trial protocol.
(0.07 MB DOC)

## References

[1] Naftanel MA, Harlan DM (2004). Pancreatic islet transplantation.. PLoS Med.

[2] Shapiro AM, Lakey JR, Ryan EA, Korbutt GS, Toth E (2000). Islet transplantation in seven patients with type 1 diabetes mellitus using a glucocorticoid-free immunosuppressive regimen.. N Engl J Med.

[3] Keymeulen B, Ling Z, Gorus FK, Delvaux G, Bouwens L (1998). Implantation of standardized beta-cell grafts in a liver segment of IDDM patients: graft and recipients characteristics in two cases of insulin-independence under maintenance immunosuppression for prior kidney graft.. Diabetologia.

[4] Hering BJ, Kandaswamy R, Harmon JV, Ansite JD, Clemmings SM (2004). Transplantation of cultured islets from two-layer preserved pancreases in type 1 diabetes with anti-CD3 antibody.. Am J Transplant.

[5] Ricordi C, Inverardi L, Kenyon NS, Goss J, Bertuzzi F, Alejandro R (2005). Requirements for success in clinical islet transplantation.. Transplantation.

[6] Maleux G, Gillard P, Keymeulen B, Pipeleers D, Ling Z (2005). Feasibility, safety, and efficacy of percutaneous transhepatic injection of beta-cell grafts.. J Vasc Interv Radiol.

[7] Ryan EA, Paty BW, Senior PA, Bigam D, Alfadhli E (2005). Five-year follow-up after clinical islet transplantation.. Diabetes.

[8] Rother KI, Harlan DM (2004). Challenges facing islet transplantation for the treatment of type 1 diabetes mellitus.. J Clin Invest.

[9] Jansson L, Carlsson PO (2002). Graft vascular function after transplantation of pancreatic islets.. Diabetologia.

[10] Pinkse GG, Bouwman WP, Jiawan-Lalai R, Terpstra OT, Bruijn JA, de Heer E (2006). Integrin signaling via RGD peptides and anti-beta1 antibodies confers resistance to apoptosis in islets of Langerhans.. Diabetes.

[11] Sasaki TM, Gray RS, Ratner RE, Currier C, Aquino A (1998). Successful long-term kidney-pancreas transplants in diabetic patients with high C-peptide levels.. Transplantation.

[12] Nanji SA, Shapiro AM (2004). Islet transplantation in patients with diabetes mellitus: choice of immunosuppression.. BioDrugs.

[13] Keymeulen B, Gillard P, Mathieu C, Movahedi B, Maleux G (2006). Correlation between beta cell mass and glycemic control in type 1 diabetic recipients of islet cell graft.. Proc Natl Acad Sci U S A.

[14] Atkinson MA, Eisenbarth GS (2001). Type 1 diabetes: new perspectives on disease pathogenesis and treatment.. Lancet.

[15] Roep BO (2003). The role of T-cells in the pathogenesis of Type 1 diabetes: From cause to cure.. Diabetologia.

[16] Uchikoshi F, Yang ZD, Rostami S, Yokoi Y, Capocci P (1999). Prevention of autoimmune recurrence and rejection by adenovirus-mediated CTLA4Ig gene transfer to the pancreatic graft in BB rat.. Diabetes.

[17] Bartlett ST, Schweitzer EJ, Kuo PC, Johnson LB, Delatorre A, Hadley GA (1997). Prevention of autoimmune islet allograft destruction by engraftment of donor T cells.. Transplantation.

[18] Tyden G, Reinholt FP, Sundkvist G, Bolinder J (1996). Recurrence of autoimmune diabetes mellitus in recipients of cadaveric pancreatic grafts.. N Engl J Med.

[19] Bosi E, Braghi S, Maffi P, Scirpoli M, Bertuzzi F (2001). Autoantibody response to islet transplantation in type 1 diabetes.. Diabetes.

[20] Jaeger C, Brendel MD, Hering BJ, Eckhard M, Bretzel RG (1997). Progressive islet graft failure occurs significantly earlier in autoantibody-positive than in autoantibody-negative IDDM recipients of intrahepatic islet allografts.. Diabetes.

[21] Jaeger C, Brendel MD, Eckhard M, Bretzel RG (2000). Islet autoantibodies as potential markers for disease recurrence in clinical islet transplantation.. Exp Clin Endocrinol Diabetes.

[22] Slover RH, Eisenbarth GS (1997). Prevention of type I diabetes and recurrent beta-cell destruction of transplanted islets.. Endocr Rev.

[23] Verge CF, Stenger D, Bonifacio E, Colman PG, Pilcher C (1998). Combined use of autoantibodies (IA-2 autoantibody, GAD autoantibody, insulin autoantibody, cytoplasmic islet cell antibodies) in type 1 diabetes: Combinatorial Islet Autoantibody Workshop.. Diabetes.

[24] Martin S, Wolf-Eichbaum D, Duinkerken G, Scherbaum WA, Kolb H (2001). Development of type 1 diabetes despite severe hereditary B-lymphocyte deficiency.. N Engl J Med.

[25] Palmer JP, Fleming GA, Greenbaum CJ, Herold KC, Jansa LD (2004). C-peptide is the appropriate outcome measure for type 1 diabetes clinical trials to preserve beta-cell function: report of an ADA workshop, 21–22 October 2001.. Diabetes.

[26] Shapiro AM, Ricordi C, Hering BJ, Auchincloss H, Lindblad R (2006). International trial of the Edmonton protocol for islet transplantation.. N Engl J Med.

[27] Bouma GJ, van der Meer-Prins PM, van Bree FP, van Rood JJ, Claas FH (1992). Determination of cytotoxic T-lymphocyte precursor frequencies using europium labeling as a nonradioactive alternative to labeling with chromium-51.. Hum Immunol.

[28] Roep BO, Stobbe I, Duinkerken G, van Rood JJ, Lernmark A (1999). Auto- and alloimmune reactivity to human islet allografts transplanted into type 1 diabetic patients.. Diabetes.

[29] Roep BO (1996). T-cell responses to autoantigens in IDDM. The search for the Holy Grail.. Diabetes.

[30] Roep BO, Kallan AA, Duinkerken G, Arden SD, Hutton JC (1995). T-cell reactivity to beta-cell membrane antigens associated with beta-cell destruction in IDDM.. Diabetes.

[31] Terasaki PI, Cai J (2005). Humoral theory of transplantation: further evidence.. Curr Opin Immunol.

[32] Decochez K, Tits J, Coolens JL, Van Gaal L, Krzentowski G (2000). High frequency of persisting or increasing islet-specific autoantibody levels after diagnosis of type 1 diabetes presenting before 40 years of age. The Belgian Diabetes Registry.. Diabetes Care.

[33] Patel R, Terasaki PI (1969). Significance of the positive crossmatch test in kidney transplantation.. N Engl J Med.

[34] Strijbosch LW, Does RJ, Buurman WA (1988). Computer aided design and evaluation of limiting and serial dilution experiments.. Int J Biomed Comput.

[35] Stobbe I, Duinkerken G, van Rood JJ, Lernmark A, Keymeulen B (1999). Tolerance to kidney allograft transplanted into Type I diabetic patients persists after in vivo challenge with pancreatic islet allografts that express repeated mismatches.. Diabetologia.

[36] van Kampen CA, van de Linde P, Duinkerken G, van Schip JJ, Roelen DL (2005). Alloreactivity against repeated HLA mismatches of sequential islet grafts transplanted in non-uremic type 1 diabetes patients.. Transplantation.

[37] Roep BO (1999). Standardization of T-cell assays in Type I diabetes.. Diabetologia.

[38] Peakman M, Tree TI, Endl J, van Endert P, Atkinson MA, Roep BO (2001). Characterization of preparations of GAD65, proinsulin, and the islet tyrosine phosphatase IA-2 for use in detection of autoreactive T-cells in type 1 diabetes - Report of phase II of the second international immunology of diabetes society workshop for standardization of T-cell assays in type 1 diabetes.. Diabetes.

[39] Goudy KS, Tisch R (2005). Immunotherapy for the prevention and treatment of type 1 diabetes.. Int Rev Immunol.

[40] Sutherland DE, Gruessner RW, Gruessner AC (2001). Pancreas transplantation for treatment of diabetes mellitus.. World J Surg.

[41] Pinkse GG, Tysma OH, Bergen CA, Kester MG, Ossendorp F (2005). Autoreactive CD8 T cells associated with beta cell destruction in type 1 diabetes.. Proc Natl Acad Sci U S A.

[42] Sibley RK, Sutherland DE, Goetz F, Michael AF (1985). Recurrent diabetes mellitus in the pancreas iso- and allograft. A light and electron microscopic and immunohistochemical analysis of four cases.. Lab Invest.

[43] Sutherland DE, Goetz FC, Sibley RK (1989). Recurrence of disease in pancreas transplants.. Diabetes.

[44] Braghi S, Bonifacio E, Secchi A, Di CV, Pozza G, Bosi E (2000). Modulation of humoral islet autoimmunity by pancreas allotransplantation influences allograft outcome in patients with type 1 diabetes.. Diabetes.

[45] Petruzzo P, Andreelli F, McGregor B, Lefrancois N, Dawahra M (2000). Evidence of recurrent type I diabetes following HLA-mismatched pancreas transplantation.. Diabetes Metab.

[46] Lehmann PV, Sercarz EE, Forsthuber T, Dayan CM, Gammon G (1993). Determinant spreading and the dynamics of the autoimmune T-cell repertoire.. Immunol Today.

[47] Bougneres PF, Landais P, Boisson C, Carel JC, Frament N (1990). Limited duration of remission of insulin dependency in children with recent overt type I diabetes treated with low-dose cyclosporin.. Diabetes.

[48] Silverstein J, Maclaren N, Riley W, Spillar R, Radjenovic D, Johnson S (1988). Immunosuppression with azathioprine and prednisone in recent-onset insulin-dependent diabetes mellitus.. N Engl J Med.

[49] Eisenbarth GS, Srikanta S, Jackson R, Rabinowe S, Dolinar R (1985). Anti-thymocyte globulin and prednisone immunotherapy of recent onset type 1 diabetes mellitus.. Diabetes Res.

