# Supplementary material for: Cellular Islet Autoimmunity Associates with Clinical Outcome of Islet Cell Transplantation
Source: PLoS One. 2008 Jun 18;3(6):e2435. doi: 10.1371/journal.pone.0002435 (PMC2426735; doi:10.1371/journal.pone.0002435)
Supplement: Table S1 — Characteristics of immune reactivity and clinical parameters per patient. (0.14 MB DOC) [file pone.0002435.s001.doc]

#### Table S1. Characteristics of immune reactivity and clinical outcome per patient

|  | **Patient:** | **1** | **2** | **3** | **4** | **5** | **6** | **7** | **8** | **9** | **10** | **11** | **12** | **13** | **14** | **15** | **16** | **17** | **18** | **19** | **20** | **21** |
| --- | --- | --- | --- | --- | --- | --- | --- | --- | --- | --- | --- | --- | --- | --- | --- | --- | --- | --- | --- | --- | --- | --- |
| **Immune parameters:** |  |  |  |  |  |  |  |  |  |  |  |  |  |  |  |  |  |  |  |  |  |  |
| **ATG dosage**  (mg/kg) |  | 21.1 | 23.4 | 24.0 | 20.8 | 22.9 | 20.9 | 24.0 | 19.6 | 23.6 | 26.7 | 22.0 | 26.5 | 24.9 | 21.0 | 24.5 | 30.8 | 20.2 | 18.0 | 23.8 | 18.0 | 21.9 |
| **Tacrolimus trough level**  (median 0-12 months, ng/ml) |  | 6.0 | 5.8 | 4.1 | 7.5 | 7.0 | 9.0 | 9.0 | 9.0 | 8.7 | 8.0 | 7.0 | 8.6 | 6.0 | 9.0 | 8.7 | 10.0 | 9.0 | 10.0 | 10.3 | 8.6 | 9.1 |
| **MMF dosage**  (median 0-12 months, mg/day) |  | 2000 | 2000 | 1500 | 2000 | 2000 | 2000 | 2000 | 2000 | 2000 | 1500 | 1000 | 2000 | 1500 | 2000 | 1500 | 2000 | 2000 | 2000 | 1500 | 1250 | 2000 |
| **Pre transplant cellular autoreactivity** | GAD | - | + | + |  | - | - | - | - | + | + | + | - | - | - | - | + | + | - | - | - | - |
|  | IA-2 | + | + | + |  |  |  | - | - | + | - | + | + | + | - | - | - | - | - | - | - | - |
| **Pre transplant auto-antibodies** | ICA | - | - | - | - | - | - | - | - | - | + | + | - | - | + | - | + | - | - | + | - | - |
|  | GAD | + | - | - | + | - | + | + | + | - | - | + | + | - | + | - | + | + | - | + | - | - |
|  | IA-2 | - | - | + | - | + | - | + | - | - | - | + | - | + | + | + | + | + | - | - | - | - |
| **Post transplant cellular autoreactivity** *(wk of 1st appearance)* | GAD | - | - | - | - | - | - | - | + *(4)* | - | - | - | - | - | - | + *(6)* | - | - | - | - | - | - |
|  | IA-2 | + *(2)* | + *(5)* | +*(6)* |  |  |  | + *(6)* | + *(6)* | +*(18)* | - | + *(2)* | - | - | - | + *(2)* | - | - | - | - | +*(15)* | - |
| **Post transplant auto-antibody seroconversion** |  | = | ↑ | ↑ | = | ↑ | = | = | = | = | ↓ | = | = | = | ↓ | = | = | = | = | = | = | = |
| **Post transplant cellular alloreactivity** |  | - | + | - |  | - | + | + | - | - | - | + | - | + | + | + | - | - | - | - | + | + |
| **Transplant parameters:** |  |  |  |  |  |  |  |  |  |  |  |  |  |  |  |  |  |  |  |  |  |  |
| **Number of transplants** |  | 2 | 2 | 2 | 1 | 1 | 2 | 1 | 2 | 2 | 2 | 1 | 2 | 1 | 1 | 1 | 2 | 2 | 1 | 2 | 2 | 1 |
| **Number of donors** |  | 6 | 8 | 6 | 4 | 5 | 3 | 3 | 6 | 6 | 5 | 5 | 9 | 10 | 6 | 6 | 9 | 9 | 3 | 10 | 9 | 2 |
| **Total injected beta cell mass**  (x10^6^ β-cells/kg) |  | 2.5 | 3.4 | 2.3 | 2.6 | 4.5 | 4.1 | 2.3 | 3.8 | 4.6 | 9.0 | 3.1 | 5.7 | 5.7 | 4.2 | 3.9 | 3.7 | 4.0 | 3.5 | 4.3 | 5.4 | 2.2 |
| **All injections ≥ 2.0x10^6^ β-cells/kg** |  | n | n | n | y | y | n | y | n | y | y | y | y | y | y | y | n | n | y | y | y | y |
| **Outcome parameters:** |  |  |  |  |  |  |  |  |  |  |  |  |  |  |  |  |  |  |  |  |  |  |
| **Insulin independence reached** |  | n | n | n | y | y | n | y | y | n | y | n | y | y | y | n | n | y | y | y | y | y |
| (weeks after transplantation) |  | n/a | n/a | n/a | 15.0 | 17.1 | n/a | 13.7 | 25.7 | n/a | 20.1 | n/a | 17.3 | 12.0 | 5.9 | n/a | n/a | 20.0 | 12.0 | 23.9 | 14.9 | 11.3 |
| **Insulin independence at one year** |  | n | n | n | y | y | n | y | y | n | y | n | y | n | y | n | n | n | n | y | y | y |
| **Plasma C-peptide level**  (AUC 0-52 weeks) |  | 46.8 | 54.7 | 6.2 | 105.0 | 89.3 | 33.2 | 108.9 | 72. 4 | 28.0 | 88.4 | 70.9 | 121.6 | 71.1 | 131.6 | 81.1 | 47.2 | 91.3 | 117.4 | 81.9 | 148.7 | 137.2 |
