# Supplementary material for: Cellular Islet Autoimmunity Associates with Clinical Outcome of Islet Cell Transplantation
Source: PLoS One. 2008 Jun 18;3(6):e2435. doi: 10.1371/journal.pone.0002435 (PMC2426735; doi:10.1371/journal.pone.0002435)
Supplement: Protocol S1 — Trial protocol. (0.07 MB DOC) [file pone.0002435.s004.doc]

# CONFIDENTIAL

**ß Cell Transplantation in Diabetes Protocol IV**

RATIONALE FOR ß CELL TRANSPLANTATION IN PATIENTS WITH EARLY STAGE DIABETIC COMPLICATIONS (NEPHROPATHY, RETINOPATHY, HYPOGLYCEMIA UNAWARENESS)

Current treatment of diabetes does not prevent episodes of abnormally high blood glucose levels. Recurrent hyperglycemia may cause chronic lesions that can lead to renal failure, blindness, renal failure, neuropathy and atherosclerosis. A major task of diabetes research consists in developing new forms of treatment which prevent or delay these disabling complications of the disease.

Transplantation of pancreatic organs can restore normoglycemia in insulin-dependent diabetes. Long-term functioning grafts are able to stop the progression of diabetic glomerulopathy and other diabetic complications and improve the quality of life. A solitary pancreas transplantation can presently, however, not be considered at an early stage of the disease given the disadvantageous risk - benefit ratio of the surgical intervention and the obligatory continuous association to immunosuppressive drugs.

Beta cell grafts can be transplanted at a much lower risk since donor tissue is tested pre-implantation and since intraportal islet cell implantation can be done with a low risk of morbidity. Human islet cell allotransplants might not require permanent immunosuppression if the outcome is similar than in rodents as culture of the donor tissue seems associated with a reduced immunogenicity (Pipeleers D et al., Diabetes Annual, 1994). In our first protocol in type 1 diabetic patients with a well-functioning kidney allograft, five out of five ß cell transplants with a history of anti-thymocyte-globulin (ATG) at the time of renal transplantation, survived for more than one year in type 1 diabetic patients who solely received the maintenance immunossuppressive therapy that was installed for a renal allograft more than one year before the ß-cell graft. Patients with surviving beta cell grafts exhibit near-normal HbA1c levels, and when transplanted with > 1.5 million beta cells/kg became totally insulin-independent (Keymeulen B et al., Diabetologia, 1998).

The clinical potential of ß-cell transplantation will of course depend on its ability to correct glucose homeostasis at an earlier stage of the disease. The present proposal aims at diabetic patients with early stage chronic complications (nephropathy and/or retinopathy) and/or hypoglycemic unawareness. This patient group will receive a temporary treatment with ATG (ATG®-Fresenius), mycophenolate mofetil and (MMF - Cell-Cept®) and tacrolimus (Prograf®).

In whole organ transplantation, ATG is given at the time of implantation in order to decrease and/or postpone acute rejection. Because this form of therapy initially results in a cytokine release syndrome that is most severe after the first infusion, ATG treatment is started one day before the ß-cell transplantation. The purpose is to avoid any deteriorating action of the cytokines on the implant. In renal transplantation, MMF prevents acute graft rejection more frequently than azathioprine (Sollinger HW et al., Transplantation, 1995; European Mycophenolate Mofetil Cooperative Study Group, Lancet ,1995). In our experience with beta cell transplantation in renal transplant patients without a history of ATG (protocol III), MMF treatment (2gr daily) was not associated with major side-effects and resulted in prolonged ß-cell graft function up to 3 months in 5 out of 5 recipients versus less than 3 weeks in the azathioprine group (protocol I; n=4 patients). MMF will be started on the day of the ß-cell transplantation.

Tacrolimus is a calcineurin-inhibitor that is successfully used in pancreatic transplantation. In this protocol a low dose will be given orally twice daily to maintain a 12 hr through concentration between 3 and 6 ng/ml. Results with tacrolimus in islet transplantation protocols are encouraging. In the recently published study by the Edmonton group, similar low doses of tacrolimus, in combination with anti-IL2 receptor antibodies and rapamycin were well tolerated and resulted in a 1 year survival of the islets in 100% of patients (Shapiro J et al, New England Journal of Medicine, 2000).

The theoretical benefits of the presently proposed protocol (IV) for the diabetic patient have to be weighed against its risks:

- It has been suggested that 25 to 45 percent of type 1 diabetic patients will, during their life-time, develop clinically evident nephropathy (Orchard TJ et al., Diabetes, 1990). Studies have associated a number of factors with the development risk of renal lesions, namely genetic susceptibility, blood pressure and glycemic control. Microalbuminuria in diabetes with a duration of less than 15 years is predictive for progressive renal disease (50-80%). ACE inhibitors lower blood pressure and dramatically reduce the risk of clinical nephropathy in Type 1 diabetic patients with microalbuminuria. However, during a 24-month follow-up, 7.2 percent of patients with microalbuminuria progressed further to clinical proteinuria despite treatment with captopril (The microalbuminuria captopril study group, Diabetologia, 1996). In a stepwise proportional hazards analysis, a higher baseline albumin excretion rate and a higher baseline glycated haemoglobin were found to be independent predictors of progression to clinical proteinuria. It has been suggested that the decline in glomerular filtration rate starts when the albumin excretion rate is higher than 100 mg/24 hrs (De Fronzo RA, Diabetes Reviews, 1995). Therefore, in this protocol, patients will be recruited with albuminuria of 100 - 1000 mg/24 hrs, despite optimal antihypertensive therapy, preferably with ACE inhibitors. In terms of natural history of diabetic nephropathy, a beneficial effect of optimal glucose control has clearly been evidenced till the stage of low-range clinical proteinuria. Evolution of retinopathy is again determined by glycemic control (DCCT Study Group, New England Journal of Medicine, 1993). In all studies the degree of glycemic control is limited by the occurence of (severe) hypoglycemias. The risk for severe hypoglycemia is the highest in patients who suffer from hypoglycemia unawareness. Therefor, in addition in this protocol preference will be given to patients who also suffer from (pre)proliferative retinopathy and/or hypoglycemic unawareness.

- Potential side-effects of protocol IV primarily relate to the immunosuppressive therapy, as no morbidity has occured yet related to the implantation procedure. During the time of immunosuppressive therapy, the patient is at risk for infections (primarily CMV). However, in ß cell preparations, that are cultured for more than 48 hrs, less than 0.1% MHC class II positive cells are detected (Keymeulen B et al., Diabetologia, 1998). Moreover, in 10 islet ß cell preparations from CMV-IgG positive donors, polymerase chain reaction test for CMV-DNA was negative (unpublished observations). In previous protocols, all CMV negative recipients, that received ß cells from CMV-IgG positive donors, remained CMV negative (n=15). For these reasons, we think that the chances of transmission of CMV along with the ß-cell implant is unlikely to occur, which contrasts with the risks by whole organ transplantation. On a weekly basis, a CMV polymerase chain reaction test will be performed on peripheral blood leukocytes for rapid detection of CMV viremia. Still Ganciclovir by mouth will be administered (3g/d) as preventive therapy for 100 days. In order to prevent infection with Pneumocystis carinii, one tablet of Bactrim Forte will be given daily for 100 days.

- The risk of cancer and post-transplantation lymphoproliferative disorder (PTLD) is primarily related to the duration and degree of immunosuppressive therapy. It is considered to be low when immunosuppressive therapy is only administered short-term; in patients with surviving beta-cell grafts, immunosuppressive therapy will be tapered down after one year. As EBV-negative recipients are more prone to PTLD, we will exclude these patients.

- Finally, the induction immunosuppressive therapy in protocol IV should not endanger renal function. Renal damage is an important potential side-effect of tacrolimus therapy that can, however, be minimized by reducing the dose and length of administration.

AIM OF THE STUDY

To examine whether temporary immunsuppression with ATG, tacrolimus and MMF allows prolonged survival of ß cell allografts in Type 1 diabetic patients with early chronic complications of diabetes

RECIPIENT GROUP

**Criteria for selection**

Type 1 insulin-dependent diabetic patients in relatively good general condition

- age 18-65 years

- non-smoker before transplantation

- body weight < 80 kg

- C-peptide < 0.15 nmol/l (<0.45 µg/l) 6 min. after glucagon IV (1mg) (glycemia > 180 mg/dl)

- responsible physicians should aim to reduce mean systolic blood pressure to less than 130 mmHg and mean diastolic blood pressure less than 85 mmHg, when measured at home with ambulatory BP monitoring

- plasma creatinine < 2 mg/dl

- albuminuria <1000 mg/24 hrs

- normal liver function
- no history of thrombosis or pulmonary embolism
- no history of malignancy, tuberculosis or chronic viral hepatitis
- no history of any other serious illness which could be relevant for the protocol

- no HLA antibodies

- EBV antibody positive
- HIV 1 & 2 antibody negative

- CMV IgM negative; IgG negative or positive

- cooperative and reliable patient giving informed consent by signature; the patient should be informed in sufficient detail on the content and procedure of the protocol, indicating potential risks of intervention and of immunosuppressive therapy; the patient should also be informed that withdrawal of immunosuppressive therapy in patients with persistent plasma C-peptide positivity may result in subsequent loss of ß-cell graft function; the ß cell implant should be identified as a clinical trial.

In candidates that full-fill all inclusion criteria, non-inherited maternal antigens (NIMA) will be identified by voluntary blood sampling of their mother/father.

**Further ranking by Eurotransplant:**

# A Status of chronic complications

1 early chronic complications of diabetes:

- patients with albuminuria (defined as 100-1000 mg/ 24 hrs on 3 separate determinations > 1 month and outside an episode of illness) and/or retinopathy (preproliferative or proliferative) will receive first priority for islet cell transplantation.

2 no early chronic complications as defined above

- patients without chronic complications can also be screened but will only be transplanted in function of ß-cell availability.

*B ABO blood group:*

1 bloodgroup AB

2 bloodgroup A

3 bloodgroup B

4 bloodgroup O

# C Islet cell antibody status

1 islet cell antibody negative

2 islet cell antibody positive

**Number of patients**

Patients in the Eurotransplant region, Finland and Malmö will be recruited by participating clinical centers on the basis of the selected criteria. Participating physicians transmit a list of patients with complete information, clinical and biological data, informed consent to the central unit (Drs B. Keymeulen and D. Pipeleers). These data will be used to select the patients, in agreement with the patient's physician. Three members at the Central Unit preserve the medical secret of the central list (Dr. D. Pipeleers, project leader; C. Hendrieckx, central unit coordinator; Dr. B. Keymeulen, clinical trial coordinator).

Severe adverse events associated with treatment will be reported immediately to the PMG.

Islet graft characteristics

Each patient will receive subsequent islet grafts of minimally 0.5 million beta cells /kg per implant. Each implant will be derived from one pancreatic segment that is processed within 3 to 5 days before implantation. After 3- 5 days of culture the implant should contain more than 30 percent endocrine cells, more than 20 percent insulin-positive cells, less than 10 percent damaged cells, less than 5 percent exocrine cells, less than 5 percent MHC-class II positive cells. Subsequent implantations will only be done in patients that are plasma C-peptide positive at the time of subsequent implantation. The aim is to implant in total minimally 2.0 million beta-cells/kg b.w.

Exclusion criteria will focus on the presence or likelihood of infection by HIV-1 and HIV-2, hepatitis A, B and C virus, as reported by the donor centre. These measurements will be repeated in the implantation centre as well as a serology for HTLV-1 and lues; these results will only be available after beta-cell transplantation has occurred. For each implant, culture medium will be screened for the presence of fungi and yeast, mycoplasma and for aerobic bacteria using classical culture methods.

Donors, that fulfill the quality control criteria (see above) should be matched with the recipient for ABO blood group system. The influence of HLA matching/mismatching will only be examined retrospectively. Before transplantation, a cross-match will be done that should be negative. In addition to HLA-typing, donors will be genotyped for IDDM risk alleles.

##### INJECTION OF ISLET CELL GRAFTS INTO THE LIVER

Islets will be injected in a branch of the portal system in the liver. Before insertion an ultrasound examination of the liver will be performed to exclude anatomic abnormalities. After sedation, a percutaneous transhepatic approach will be used to gain access to the portal vein under ultrasound guidance.

Once access is confirmed, a Seldinger technique is used to place 4-5 Fr catheter within the portal vein (branch). The final position of the catheter is documented with a test injection of contrast medium. The total amount of intravenous contrast will be small.

The final islet preparation (less than 2ml) is suspended in 60-100ml of medium HAM-F10 that contains 0.5 percent human albumin and is infused over a period of 3-5 minutes. The catheter is removed after all islet cells have been injected. The whole procedure will be performed in the radiology department under sterile conditions.

Doppler ultrasonography of the portal vein and liver-function tests will be performed within 24 hours after transplantation.

## IMMUNOSUPPRESSIVE TREATMENT

### In this section, the day of first beta-cell implant will be named day 0

Day –4:

*1 Anti-thymocyte globulin (ATG).* Placement of central venous line by anaesthesiologist.

A test dose of *ATG* (Fresenius®; 5mg over 10 min) is given via a central vein (CV). If no major side effects occur within 2 hours, *methylprednisolone* (500 mg iv over 30 min.) and promethazine (10 mg iv in shot) are given 60 min before administration of ATG (Fresenius®; 9 mg/kg over 12 hours via CV).

- Start oral *MMF* (Cell-Cept®) 2 x 1 gr daily ; reduce dose if WBC count = 3000/mm3.

Day –3 and -2:

*- ATG* (Fresenius®; 3 mg/kg over 12-24 hours via CV ) is administered except when trombocyte count is less than 50.000/mm3 or T-lymphocyte count < 50/ mm3.

- *MMF* is continued at the dose that is well tolerized; reduce dose if WBC count = 3000/mm3.

Day -1:

*- ATG* (Fresenius®; 3 mg/kg over 12-24 hours via CV ) is administered except when trombocyte count is less than 50.000/mm3.

- *MMF* is continued at the dose that is well tolerized; reduce dose if WBC count = 3000/mm3.

Day 0:

- First implant of ß cells ; *methylprednisolone* (500 mg iv over 30 min.) is administered at -3 hrs

*- ATG* (Fresenius®; 3 mg/kg over 12-24 hours via CV ) is administered except when trombocyte count is less than 50.000/mm3.

- *MMF* is continued at the dose that is well tolerized; reduce dose if WBC count = 3000/mm3.

Day 1:

- *ATG* (Fresenius®; 3 mg/kg over 12-24 hours via CV ) is administered except when trombocyte count is less than 50.000/mm3 or T-lymphocyte count is less than 50/mm3. .

- *MMF* is continued at the dose that is well tolerized; reduce dose if WBC count = 3000/mm3.

Day 2:

- *ATG* (Fresenius®; 3 mg/kg over 12-24 hours via CV ) is administered except when trombocyte count is less than 50.000/mm3 or T-lymphocyte count is less than 50/mm3.

- Start oral tacrolimus (Prograf®) 2 x 0.05mg/kg; subsequently adjust dose to maintain a through concentration at 12 hours of 8-10 ng/ml

- *MMF* is continued at the dose that is well tolerized; reduce dose if WBC count = 3000/mm3.

Day 3:

- *ATG* (Fresenius®; 3 mg/kg over 12-24 hours via CV ) is administered except when trombocyte count is less than 50.000/mm3 or T-lymphocyte count is less than 50/mm3.

- Tacrolimus is continued (through concentration at 12 hours of 8-10 ng/ml

- *MMF* is continued at the dose that is well tolerized; reduce dose if WBC count = 3000/mm3.

Day 4 -…:

- *MMF* is continued at the dose that is well tolerized; reduce dose if WBC count = 3000/mm3.

- Tacrolimus is continued (through concentration at 12 hours of 8-10 ng/ml the first 12 weeks and 6-8ng/ml 12-52 weeks PT)

**In this section the subsequent beta-cell implants will be called day 0:**

Day 0:

- *MMF* is continued at the dose that is well tolerized; reduce dose if WBC count = 3000/mm3.

- Tacrolimus is continued (through concentration at 12 hours of 8-10 ng/ml)

- Implant of ß cells ; *methylprednisolone* (500 mg iv over 30 min.) is administered at -3 hrs.

Day 1 -…..:

- *MMF* is continued at the dose that is well tolerized; reduce dose if WBC count = 3000/mm3.

- Tacrolimus is continued (through concentration at 12 hours of 8-10 ng/ml the first 12 weeks and 6-8ng/ml 12-52 weeks PT).

## INSULIN TREATMENT

Blood glucose levels should be kept between 70 and 180 mg%. The patient will be on intravenous insulin from the day 0 till day 7 posttransplant with at least hourly control of glycemia. Afterwards, minimally 4 daily insulin injections are recommended. An insulin pump can be used. Self-monitoring, minimally 7 times daily during the first 6 weeks posttransplantation, will be performed with a device with internal memory (i.e. One Touch II (Life Scan))

## ANTI-INFECTIOUS TREATMENT

Oral ganciclovir (1g three times per day) and Bactrim Forte (one tablet per day) is administered during 100 days after each beta-cell implant. Until 12 weeks posttransplantation of each ß cell implant, a CMV polymerase chain reaction test will be performed (preferentially weekly) on peripheral blood leukocytes for rapid detection of CMV viremia.

### ANTI-THROMBOTIC TREATMENT

Oral Aspirin (100 mg per day) and subcutaneous low-molecular weight heparins (preventive dose) are administered till day 7 PT after each beta-cell implant, unless clinical signs of bleeding and/or thrombocyte count is less than 50.000/mm^3^

Aspirin is stopped after the first islet cell injection if a second transplant is planned.

MONITORING OF PATIENTS

*Islet graft function* *after 1st and 2nd ß cell implant*

(addendum 1: blood sampling from day 0 until day 420 posttransplantation (PT))

- Plasma glycemia, C-peptide (optional plasma insulin, proinsulin (split), glucagon and IAPP levels):
- Basal levels (fasting) daily for 14 days, weekly up to 6 weeks PT; bi- monthly between 6 and 30 weeks PT and monthly between 30 and 60 weeks PT if C-peptide positive
- Following intravenous glucagon at 6 weeks PT; at 12, 24, 48 wks PT if C-peptide positive
- Urinary C-peptide daily for 14 days, then weekly up to 6 weeks PT; bi-monthly between 6 and 30 weeks PT and monthly between 30 and 60 weeks PT if C-peptide positiv
- Fructosamine weekly up to 6 weeks PT; bi-monthly between 6 and 30 weeks PT and monthly between 30 and 60 weeks PT if C-peptide positive
- HbA1c bi-monthly up to 6 weeks PT; bi-monthly between 6 and 30 weeks PT and monthly between 30 and 60 weeks PT if C-peptide positive

*Immune reactivity* *before and following transplantation*

(addendum 1: blood sampling from day 0 till day 420 PT)

- Lymphocyte subpopulations in peripheral blood

- T cell responses in peripheral blood

- Islet cell antibodies, alloantibodies in serum

- Cytokines in plasma

#### General monitoring

(addendum 1: blood sampling from day 0 till day 420 PT)

- Currently used parameters, with particular attention for liver and kidney function, virus serology, bone marrow function, tacrolimus trough levels.

On a weekly basis (till 12 weeks PT of 2nd ß cell implant), a CMV polymerase chain reaction test will be performed on peripheral blood leukocytes for rapid detection of CMV viremia .

- An ultrasound of liver is done before transplantation and at 1, 2, 6, 12, 24, 52, 104, 156, and 208 wks PT.

- A magnetic resonance of the liver and portal vein is done before transplantation and at 12, 24, 52, 104, 156, and 208 wks PT.

- A chest X-ray is done before transplantation and at 12, 24, 52, 104, 156, and 208 wks PT.

- A fundoscopy is done before transplantation and at 12, 24, 52, 104, 156, and 208 wks PT.

- An EMG of the lower limbs is done before transplantation if not done < 3yrs before.-A doppler ultrasonography of lower extremity and carotid arteries is done before transplantation if not done < 3yrs before.

- An exercise ECG or MIBI is done before transplantation if not done < 3yrs before.

- A Cr-51-EDTA GFR is done before transplantation and at 52, 104, 156, and 208 wks PT.

- A coloscopy is done before transplantation if family history of polyps or carcinoma or patient > 50yr.

- An examination by gynecologist (women > 40yr) and dermatologist is done before transplantation and at 52, 104, 156, and 208 wks PT.

- An examination by stomatologist is done before transplantation.

- An ambulatory blood pressure monitoring is done before transplantation and at 52, 104, 156, and 208 wks PT. (optional if no micro-albuminuria)

- Patients will be carefully instructed by their treating physician on the rationale and practice of the follow-up, including teaching on self-adjustment of insulin dose, diet, self-monitoring and control of body weight.

RESULTS

Quality control tests of islet graft and clinical biologic tests of donor/recipient will be carried out at the central unit or selected expert laboratories. Results and samples will be stored at the central unit. Results are accessible to the investigators of the trial.

## IMPLANTATION CENTERS

The implantations planned in this protocol will be carried out in the university hospitals in Brussels and Leuven, for logistic (close to central unit) and medical reasons (experience of team with implantation technique).

The study will be conducted by active participants of *ß Cell Transplant*, who have fulfilled the criteria of strong commitment and reliable collaboration of good quality. The PMG can ask participants to serve as consultant for particular issues. The practical aspects at the selected implantation center will be organized together with local personnel.

Islet graft recipients are not necessarily patients of the implantation center; they are selected from the waiting list by the trial team and can be invited to travel to the implantation center where the trial occurs for which they are selected. The implantation center and the patients should formally agree with this procedure.

Patients with a health insurance in Belgium will have no additional medical costs during the trial. For other patients, medical costs will be covered by the clinical center that has recruited the patient according to local regulations. Follow-up costs (treatment and hospital activities) should be covered by the clinical center where the patients are followed. The central unit covers the costs of the islet graft quality control tests and of the clinical biologic tests that are performed in selected expert laboratories (addendum 2). The responsible physician of each implantation center should be sufficiently covered for all potential liability costs associated with the procedure and/or implanted human cells.

MANAGEMENT OF THE CLINICAL TRIAL

The trial team is responsible for setting-up, guiding and controlling the clinical trial, interpreting the results and deciding on the release of information. It informs the management group which serves as an advisory committee. To this aim, the committee can take additional advice from experts inside or outside the network. The central unit is responsible for the daily management, for the contacts with the implantation center and associated team. The protocol has to be approved by the local ethical committee.

Protocol and data are handled confidentially by the central unit and the trial team. The participants in the network are informed about the plans and guidelines; within 3 months after termination of the protocol, they will receive an outline of the results.

Publications and any other release of information (i.e. press) will only occur after proposal of the principal investigator in the trial and agreement by the expert committee. Authorships are decided according to the guidelines Diabetologia, 37, A10, 1994.

ABBREVIATIONS AND CLARIFICATIONS

Allograft: tissue or cells transplanted into a genetically nonidentical member of the same species.

CMV: cytomegalic virus

EBV: Epstein-Barr virus

Fructosamine: glycoated serum protein, expresses metabolic control

GAD: glutamic acid decarboxylase, enzyme present in islet cells

Glucagon: homone secreted by a population of islet cells

H.L.A.: human leukocyte antigens which code the M.H.C.

HbA1c: hemoglobin A1c, expresses metabolic control

HIV: human immunodeficiency virus

M.H.C.: major histocompatibilty complex, part of the human genome that regulates the immune reactivity to grafted tissue

MMF: mycophenolate mofetil, immunosuppressive drug

Plasma C-peptide: reflects endogenous insulin secretion
